# *Citrus* Essential Oils (CEOs) and Their Applications in Food: An Overview

**DOI:** 10.3390/plants9030357

**Published:** 2020-03-11

**Authors:** Himashree Bora, Madhu Kamle, Dipendra Kumar Mahato, Pragya Tiwari, Pradeep Kumar

**Affiliations:** 1 Department of Forestry, North Eastern Regional Institute of Science and Technology, Nirjuli 791109, India; bora.heena12@gmail.com (H.B.); madhu.kamle18@gmail.com (M.K.); 2 School of Exercise and Nutrition Sciences, Deakin University, 221 Burwood Hwy, Burwood, VIC 3125, Australia; kumar.dipendra2@gmail.com; 3 Department of Biotechnology, Yeungnam University, Gyeongsan, Gyeongbuk 38541, Korea

**Keywords:** *Citrus* essential oils (CEOs), antimicrobial, microencapsulation, food packaging and preservation

## Abstract

*Citrus* is a genus belonging to the Rutaceae family and includes important crops like orange, lemons, pummelos, grapefruits, limes, etc. *Citrus* essential oils (CEOs) consist of some major biologically active compounds like *α*-/*β*-pinene, sabinene, *β*-myrcene, *d*-limonene, linalool, *α*-humulene, and *α*-terpineol belonging to the monoterpenes, monoterpene aldehyde/alcohol, and sesquiterpenes group, respectively. These compounds possess several health beneficial properties like antioxidant, anti-inflammatory, anticancer, etc., in addition to antimicrobial properties, which have immense potential for food applications. Therefore, this review focused on the extraction, purification, and detection methods of CEOs along with their applications for food safety, packaging, and preservation. Further, the concerns of optimum dose and safe limits, their interaction effects with various food matrices and packaging materials, and possible allergic reactions associated with the use of CEOs in food applications were briefly discussed, which needs to be addressed in future research along with efficient, affordable, and “green” extraction methods to ensure CEOs as an ecofriendly, cost-effective, and natural alternative to synthetic chemical preservatives.

## 1. Introduction

The genus *Citrus* belongs to family Rutaceae, which includes about 140 genera and 1300 species. As a diverse tropical fruit, citrus combines species like *Citrus sinensis* (Orange), *Citrus reticulata* (Mandarin), *Citrus aurantifolia* (Limes), *Citrus limon* (Lemon), *Citrus paradisi* (Grapefruit), *Citrus bergamia* (Bergamot), *Citrus junos* (Yuzu), and *Citrus japonica* (Kumquat) [1,2]. The *Citrus* species originated from the Himalayan foothills of Northern India, Northern Myanmar, Southern China, and Southeast Asia [3,4]. Later, these fruits spread to the other parts of the world, becoming the world’s highest value fruit crops [5]. Besides its use as a condiment, *Citrus* is also used as a part of many sweet delicacies in the European countries. *Citrus* is a major component of many savory dishes, such as pan-roasted chicken with orange-brandy sauce or pork tenderloin with blood oranges. These *Citrus* fruits have a very well-inscribed nutritional value, along with high levels of elemental bioactive compounds, viz., phenols, flavonoids, limonoids, essential oils (EOs), and vitamins, particularly vitamin C and carotenoids, which have numerous health benefits. Such components give this fruit a particular flavor and aroma, leading to a more balanced and tastier diet. Above all, *Citrus* is beneficial for curing and preventing many diseases [6,7]. Several studies showed the various beneficial activities of *Citrus* and its constituents like antioxidant, antimicrobial, anti-inflammatory, insecticidal properties. *Citrus* has also shown to be beneficial in reducing the chances of developing mental health diseases, like anxiolytic against anxiety and anticholinesterase against Alzheimer’s, etc. [8]. *Citrus* fruits contain several important secondary metabolites, such as ascorbic acid, flavanones, phenolics, and pectin, which are recognized to have antioxidants properties beneficial for human health [9]. In addition, *Citrus* flavonoids are used against free radicals and possess anti-inflammatory properties and are used to reduces the brain and degenerative diseases.

**Figure 1:**
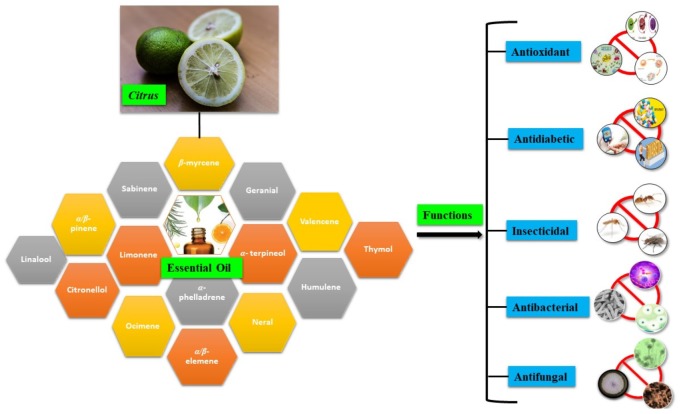
*Citrus* essential oils (CEOs) and their various functions.

Above all, the different plant parts (leaves, flowers, fruits, and peels) of *Citrus* are rich sources of EOs and other beneficial nutrients, such as vitamin B9, vitamin C, potassium, flavonoids, coumarins, pectin, and dietary fibers [10]. *Citrus* essential oils (CEOs) exhibit antioxidant, antidiabetic, insecticidal, antifungal, and antibacterial properties (Figure 1), which have important applications in the pharmaceutical, sanitary, cosmetic, agricultural, and food industries [11,12]. CEOs have a wide spectrum of applications ranging from applications in cosmetics, textiles, and pharmaceuticals to food formulations [13]. Due to their rich, pleasant, and refreshing aroma, CEOs are also used as room fresheners and disinfectants [2]. The pharmaceutical formulation prepared by adding β-cyclodextrin with orange EO (*Citrus sinensis* L.) and compounds of lemon (*Citrus limon*) helps reduce the chances of Alzheimer’s disease [14].

Nowadays, food safety has become a fundamental concern for both consumers and food industries due to the increasing awareness and concerns regarding the effects of various food components on health. Natural and organic compounds have gained significant importance for their applications in foods, as they are beneficial to health with little or no side effects, cost-effective, and environmentally friendly compared to nonorganic synthetic compounds. Therefore, plant-derived natural antimycotics have become ideal alternatives to commercial synthetic chemical preservatives for improving food quality and safety [15,16,17]. In this regard, among the plant essential oils, CEOs have drawn more attention because of their broad-spectrum insecticidal, antibacterial, and antifungal properties along with their high yields, aromas, and flavors [18,19,20,21]. Furthermore, CEOs have wide applications in food formulations, packaging, and preservation to ensure food quality and safety [22]. Therefore, this review focused on the different extraction, purification, and detection methods of EOs from various *Citrus* species, their composition, and possible applications in food safety, packaging and preservation.

## 2. Extraction, Purification and Detection Methods for *Citrus* Essential Oils (CEOs)

The extraction methods of various chemicals, along with EOs from *Citrus* and *Citrus* waste, are linked to energy consumption and CO_2_ emissions, which impact on the environment and cost. Therefore, proper and efficient extraction methods can achieve the maximum extraction, manage waste, and lead to the valorization of the byproduct [23,24]. The conventional methods of extraction viz., Soxhlet extraction, maceration, infusion, solid-liquid extraction (SLE), and liquid-liquid extraction (LLE), require greater extraction time and consume high energy, and sometimes may even require toxic solvents. In addition, the high operating temperature during the conventional extraction process may damage the heat-labile active compounds present in the extract [25]. Therefore, the current focus and requirements with extraction methods are shifting toward green extraction, which is ecofriendly with lower energy consumption and CO_2_ emissions [26]. For example, with liquid-liquid extraction (LLE), the use of biorenewable solvents based on various alkane diols have been assessed as ecofriendly substitutes to separate bioactive terpenoids from terpenes to obtain EOs during downstream processing [27]. Pourbafrani et al. [28] evaluated the emissions of greenhouse gas (GHG) associated with the biorefinery of *Citrus* processing waste (CPW), and found that replacing gasoline with ethanol (E85) in vehicle fueling and methane with natural gas, methane, for electricity production reduced GHG emissions by 134% and 77%, respectively. The modern and nonconventional techniques may require high energy to run the extraction process, such as critical pressure, supercritical fluid (liquefied CO_2_), temperature, uninterrupted electricity supply, pumps, pressure containers, and highly sealed vessels, high maintenance, microwave, and ultrasound generating equipment. However, these methods require less solvent and provide a better quality extract, along with an enhanced extraction yield within a short extraction period [26]. Furthermore, modern extraction methods are still limited to laboratory research, and industrial applications are still to be realized.

Some of the green extraction methods include supercritical fluid extraction (SFE), steam explosion, ultrasound-assisted extraction (UAE), and microwave-assisted extraction (MAE) [29]. Among these, UAE and MAE are most widely used extraction methods for essential oils and other natural products from *Citrus* species (Table 1). These methods are easy and quick to operate with lower CO_2_ emissions and energy consumption [30,31,32,33]. Similarly, supercritical fluids are nontoxic, nonflammable, and operate at low and moderate temperatures and pressures without forming chemical residue [34]. Moreover, the combinations of UAE, along with some innovative techniques like the microwave technique, instant-controlled pressure drop technique (DIC), and SFE, were found to be the most promising hybrid techniques [31]. Besides this, microwave-assisted hydrodistillation (MAHD) has also been applied to extract EOs from wet citrus peel waste [35]. In addition, microwave hydrodiffusion and gravity (MHG) is superior to conventional methods [36], as this method is solvent-free with short extraction time and works under the effect of microwaves and the Earth’s gravity [4,37]. Solvent-free extraction methods using microwave and ultrasound techniques have been successfully applied to extract EOs through hydrothermal processing [38]. Furthermore, cavitation-based extraction (CE) methods enhance the extraction quality and yield of the extract and reduce the extraction time [26]. Cavitation-based extraction (CE) methods include UAE, negative pressure cavitation (NPC) extraction, and hydrodynamic cavitation extraction (HCE). HCE is generally achieved by pumping a liquid through Venturi tubes or orifice plates which causes pressure gradient in the flow that leads to the generation, growth, and collapse of microbubbles [39]. Real-scale applications of HCE are applied to manage waste orange peels [40], as well as process other food waste [41]. HC-based processes have shown enhanced performance, yields and straightforward scalability [39,42]. In addition to this, a green and zero-energy consuming procedure based on solar energy using solar hydrodistillation (SSD) has been developed to extract essential oils [43].

The extracted compounds from *Citrus* processing waste contain a complex mixture of phytochemicals, which require further purification and refinement. The basic purification methods include column chromatography, High-Speed Counter-Current Chromatography (HSCC), High-Performance Liquid Chromatography (HPLC), and some solvent combinations, such as hexane: n-butanol, ethyl acetate: hexane, butanol: water, and chloroform: methanol, etc. [4]. After purification, the EOs present in the extract can be detected and quantified by several methods, such as Gas Chromatography-Mass Spectrometry (GC-MS), Gas Chromatography-Flame Ionization Detector (GC-FID), Nuclear Magnetic Resonance (NMR), Attenuated Total Reflection Fourier-Transform Infrared Spectroscopy (ATR-FTIR), Ultra-High Performance Liquid Chromatography Time-of-Flight Mass Spectrometry (UHPLC-TOF-MS), etc., as shown in Table 1.

## 3. Composition of *Citrus* Essential Oils (CEOs)

**Figure 2:**
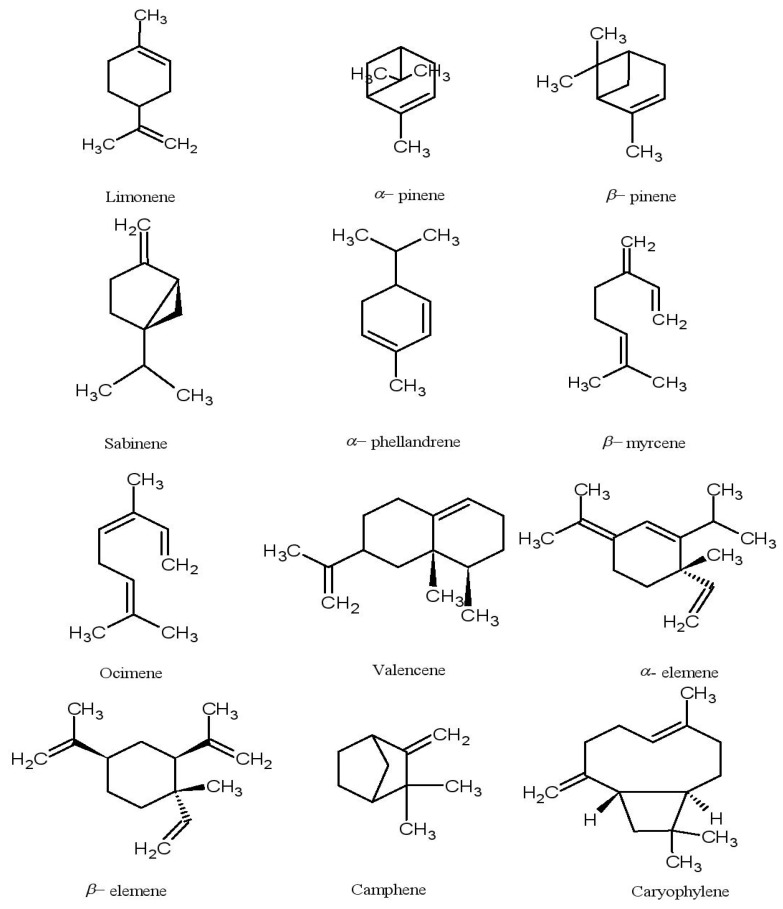

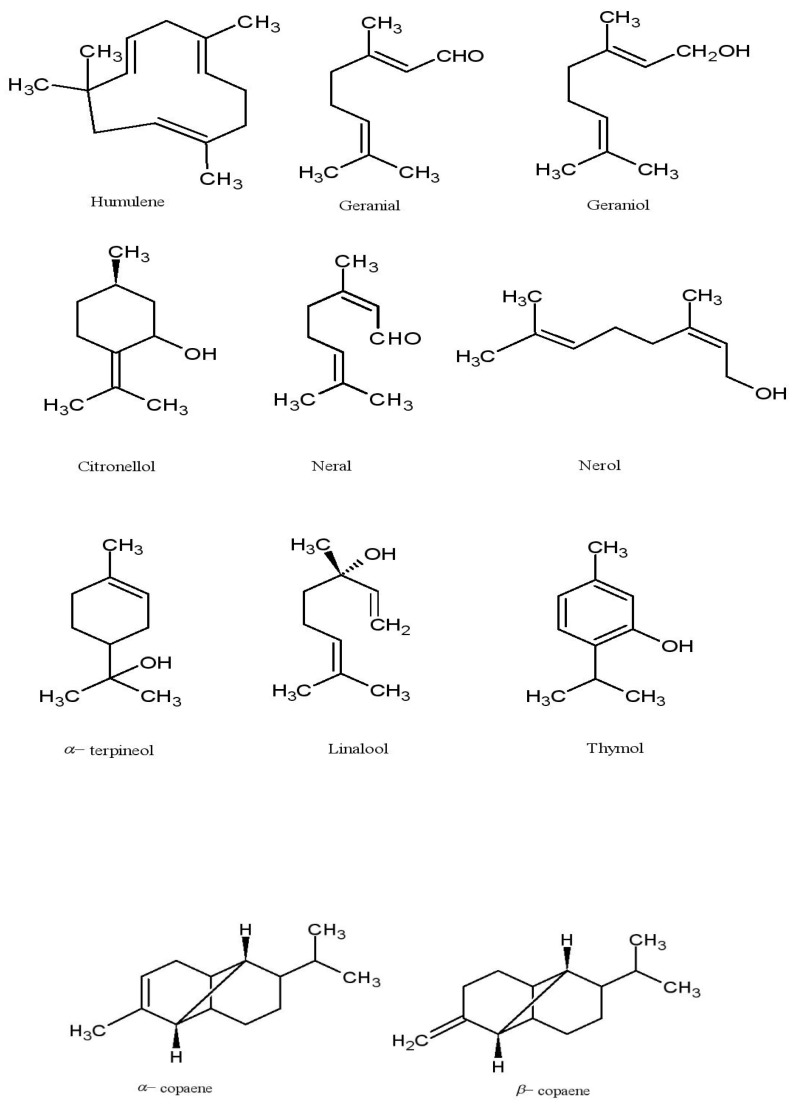
Chemical structures of some important compounds of *Citrus* essential oils (CEOs).

CEOs are a mixture of complex hydrocarbons and oxygenated derivatives of terpenoid and nonterpenoid origin consisting of functional groups such as aldehydes, alcohols, ketones, and other complex molecules, like esters and organic acids [65]. Chemically, normal edible oils and EOs are distinct from each other, as EOs are not esters of glycerides [66]. The composition of CEOs varies markedly based on variety, season, and geographic location, as well as the ripening stage of the fruit [21,67,68]. CEOs contain a wide variety of compounds, ranging from 20 to 60 compounds per CEO [69]. Of these, volatile compounds compose 85%–99%, while the remaining composition (1%–15%) consists of nonvolatile compounds. The volatile compounds contain a mixture of monoterpenes, sesquiterpenes, and sesquiterpenoids [70]. The major components are monoterpenes, which consist of two isoprene (C_5_H_8_) units and account for around 97% of the CEOs, while alcohols, aldehydes, and esters represent 1.8%–2.2% of the CEOs [21,71]. Further, among these, limonene is the main component. The concentration of limonene varies between 32% and 98% depending on the variety, for example, 32%–45% in bergamot, 45%–76% in lemon and 68%–98% in sweet orange [71]. Figure 2 depicts the chemical structures of some important compounds of *Citrus* essential oils (CEOs).

The EOs of *C. medica* in the fruit peel oil and leaf oil contain 39.37% of iso-limonene, 28.43% of erucylamide, 23.12% of citral, and 21.78% of limonene as vital components [9,72]. The EOs of *Citrus* sp. also contain an ample amount of monoterpene hydrocarbons, with limonene as the most substantial ingredient identified in the peels of *C. microcarpa* (94.0%), *C. grandis* (81.6–96.9%), and *C. aurantifolia* (39.3%). On the other hand, the peel of *C. hystrix* has sabinene as the major component of about 36.4–48.5%. Additionally, the leaves of *C. hystrix*, *C. microcarpa*, and *C. grandis* contain citronellal (61.7–72.5%), linalool (56.5%), and hedycaryol (19.0%) as the core components, respectively [9]. In addition, 33 volatile compounds were identified in *C. aurantifolia*, among which 63.35% was *d*-limonene as the major constituent, along with 7.07% of 3,7-dimethyl-2,6-octadien-1-ol, 6.23% of geraniol, 4.35% of E-citral, 3.29% of Z-citral, and 2.25% of *β*-ocimene [73]. The EO from Kaffir lime (*C. hystrix)* was analyzed by gas chromatography-mass spectrometry (GC-MS), and it was found that *β*-pinene, sabinene, limonene, and citronellal were major constituents, with concentrations of 24.62%, 22.06%, 19.29%, and 10.58%, respectively [74]. Some of the major compounds of different CEOs and their functions are summarized in Table 2.

## 4. Applications of CEO

CEOs have a wide range of applications from cosmetics to pharmaceuticals and food formulations and packaging. However, this study focused on the applications of CEOs concerning food safety, packaging, and preservation.

### 4.1. Applications of CEO for Food Safety

Commercial antimicrobial agents are used to manage food contamination and deterioration. However, due to growing health concerns, attention has been shifted to natural antimicrobials, such as plant-based essential oils. EOs and their components possess antimicrobial and food preservative properties against a broad spectrum of pathogens [93,94,95,96,97,98]. In line with this, CEOs and their insecticidal, antibacterial, and antifungal properties for food safety aspects are discussed briefly.

#### 4.1.1. CEO as an Insecticidal Agent

Conventional chemical pesticides are effective for controlling various harmful insects. However, the continued and uncontrolled use of pesticides can lead to adaptation of such insects, leading to resistance against pesticides [99,100]. Chemical-based pesticides always have a perishable impact on the environment, which threatens the entire food web and indirectly threatens the nontargeted organisms [101,102]. This complication has resulted in search of an alternative, environmentally friendly, nonhazardous plant-based option [74]. In this context, *Citrus* plants are known for their medicinal properties and are candidates to replace conventional chemical pesticides. Therefore, instead of a synthetic and inorganic chemical derived insecticide, bioactive compounds of *Citrus*, especially CEOs, can be used as a substitute [103,104].

Many studies have shown that EOs of *Citrus* sp. possess remarkable insecticidal activity. EOs from the peel of *Citrus* sp. *C. maxima* Merr. (pummelo), *C. reticulata* Blanco, *C. suncris* Linn, *C. sinensis* Linn, and *C. hystrix* were tested against the female cattle tick (*Boophilus microplus*), where the EO of *C. reticulata* showed highest acaricidal activity [105]. Similarly, the EO of sweet orange (*Citrus sinensis*) was tested against larvae and pupae of housefly (*Musa domestica*), and it was observed that the lethal concentration (LC_50_) of the EO against larvae varied between 3.93–0.71 µL/cm^2^ and 71.2–52.6 µL/L, respectively, during contact toxicity and fumigation bioassays. On the other hand, the rate of percentage inhibition of the oil varied between 27.3%–72.7% and 46.4%–100%, respectively, for contact toxicity and fumigation assay against housefly pupae [101]. In addition, EOs from plants like *C. bergamia* (bergamot), *Cymbopogon martini* (palmarosa grass), *Vetiveria zizanioides* (vetiver grass), and *Juniperus virginiana* (red cedar) showed insecticidal property against the housefly (*Musa domestica*) [106].

Furthermore, citrus essential oil-nanoparticles (CEO-NPs) nanoformulation was tested against an invasive tomato borer, *Tuta absoluta*. The assay showed an impressive result for the insecticidal properties of the compounds tested. The higher mortality was observed for EO emulsion on eggs and larvae through contact toxicity assay and by EO-NPs on larvae through ingestion toxicity [107]. Besides this, analysis by fumigant toxicity, as well as the insecticidal mechanism through acetylcholinesterase (AChE) and Na/K-ATPase activities of EO from the peel of orange against *Tribolium confusum*, *Callosobruchus maculatus,* and *Sitophylus oryzae,* were investigated by Oboh et al. [108]. The results showed detrimental effects against these insects. The percentage mortality increased with an increase in concentration and exposure time, while LC_50_ decreased with exposure time. At the highest concentration of 150 µL/L, 100% mortality was observed in *T. confusum* with LC_50_ values of 38.90 µL/L, 26.92 µL/L, and 14.45 µL/L after 24 h, 48 h, and 72 h of treatment, respectively. An increase in *C. maculatus* insecticidal activity was observed for high doses 100-150 µL/L with 100% mortality and LC_50_ values of 17.78 µL/L and 10.00 µL/L after 48 h and 72 h, respectively. Similarly, for *S. oryzae,* the insecticidal activity increased after 72 h of treatment with an LC_50_ value of 29.51 µL/L. Finally, it was revealed that for *T. confusum* and *C. maculatus*, EO exhibited higher insecticidal activity as compared to *S. oryzae*. On the other hand, studies on biochemical analysis revealed that the bioactivity of EO can hinder the mechanism of acetylcholinesterase (AChE) and Na/K-ATPase. Thus, the entire experiment stipulates the positive role of orange peel EO and its effectiveness in controlling *T. confusum*, *C. maculatus and S. oryzae* [108]. Sanei-Dehkordi et al. [109] further showed that the EO from the peel of *Citrus aurantium* and *Citrus paradisi* had larvicidal activity against *Anopheles stephensi* (a mosquito vector), with LC_50_ values of 31.20 ppm and 35.71 ppm, respectively.

#### 4.1.2. CEO as an Antibacterial Agent

In the current scenario, the state of antibiotics against human pathogenic diseases is becoming very substandard because of the increasing resistance of microorganisms toward antimicrobial drugs. It is very challenging for the researcher to come up with an improvised formulation against such pathogens which can give a long-lasting solution [110]. Medicinal plants rich in phytochemical compounds are often found to have antibacterial and antifungal properties apart from commercial insecticides and have negligible side effects [111]. Researches conducted for antibacterial activity from CEOs have given a positive response among plant-based antibiotic discoveries.

Mehmood et al. [112] analyzed the antimicrobial activity of peel EO of ripened and unripened *C. limon* against four human pathogenic bacteria viz., *Escherichia coli*, *Bacillus subtilis*, *Salmonella typhimurium,* and *Staphylococcus aureus.* Zone inhibition studies were performed using disc diffusion assay, and it was found that EO from ripened peel had the highest antibacterial activity. Chen et al. [113] determined the antibacterial activity of *C. maxima* EO against human pathogenic bacteria (*E. coli*, *B. subtilis*, *S. aureus*, *P. aeruginosa*, *B. licheniformis,* and *B. altitudinis*) using minimum inhibition concentration (MIC) ranging from 475–1104 µg/mL, where the EO showed inhibition against all the strains. Further, the antibacterial activity of EO from *C. aurantium* against eight pathogenic bacteria (*S. epidermidis*, *P. aeruginosa*, *S. aureus*, *M. luteus*, *E. coli*, *S. typhimerium*, *L. monocytogenese*, and *E. faecium*) showed maximum inhibition zone ranging from 6–16 mm for all the strains at a concentration of 7 µL per disc [114]. Similarly, the peel EO of *Citrus reticulata*, *Citrus sinensis,* and *Citrus* × *sinensis* were subjected for antibacterial analysis against four pathogenic bacteria, two Gram +ve (*S. aureus and B. subtilis*) and two Gram −ve bacteria *(E. coli* and *P. multocida*), using disc diffusion assay. The highest zone of inhibition were observed as 26.16 ± 0.28 mm for *B. subtilis*, 25.50 ± 0.50 mm for *S. aureus*, 37.42 ± 0.38 mm for *E. coli,* and 35.50 ± 0.50 mm for *P. multocida* for the EO of *C. reticulata* [115]. Further, it has been observed that EO works in synergy with other biomolecules. For example, a composite of commercial citrus essential oil and chitosan (CEO-CS) was used as perseverative for *Pneumatophorus japonicus* against two test bacteria *E. coli* and *L. monocytogenes.* The antimicrobial activity was evaluated by the oxford cup method, where the inhibition zone for chitosan (CS) was 12.24 ± 1.03 mm for *E. coli* and 13.35 ± 0.79 mm for *L. monocytogenes.* On the other hand, the CEO-CS composite showed an enhanced efficacy with a diameter of 17.23 ± 1.29 mm for *E. coli* and 19.19 ± 1.27 mm for *L. monocytogenes* [116]. The antibacterial properties of CEOs and their components are summarized in Table 3.

#### 4.1.3. CEO as an Antifungal Agent

Fungal growth is one of the leading causes of food spoilage and huge economic losses [126]. Molds can colonize and spoil a wide array of foods ranging from fresh fruits and vegetables to grains and processed foods, leading to quantitative and qualitative losses [18,126,127]. Therefore, CEOs can be employed as a natural antifungal agent to minimize the fungal growth and contamination and extend the shelf life of various foods. CEOs have a broad-spectrum fungicidal activity (Table 4). The EO of *C. sinensis* was found to be active against *Aspergillus niger* [128]. Fungicidal activity in the agar medium was evaluated with EO ranging from 0.1–3.0 µg/mL, and results showed that the highest inhibition was found at 3.0 µg/mL [128]. Lemon EO (*Citrus limon*) was used to control the fungal plant pathogens attacking grapevines, namely *Eutypa* sp., *Botryospaeria dothidea,* and *Fomitiporia mediterranea*. The antifungal activity was observed for EO against all the three pathogenic fungi with the highest activity (82% inhibition) against *Eutypa* sp. and the lowest (33.1% inhibition) against *F. mediterranea* [129]. Similarly, EO from *C. reticulata* was tested for the antifungal activity against five plants pathogenic fungi viz., *A. alternata*, *R. solani*, *C. lunata*, *F. oxysporum,* and *H. oryzae*. The activity was evaluated by the poisoned food technique and the volatile activity assay. The EO gave better results in volatile activity assay, where MIC was 0.2 mL/100mL for *A. alternata*, *R. solani*, and *C. lunata,* while in poisoned food technique, MIC was found to be >0.2 mL/100mL for *F. oxysporum* and *H. oryzae*. Besides this, spore formation was also completely inhibited at 0.2 mL/100mL except for *C. lunata* and *H. oryzae* [130]. Furthermore, CEOs from four cultivars of *Citrus* namely *C. aurantium*, *C. limon*, *C. reticulata,* and *C. sinensis* were tested against *Candida albicans* and *Aspergillus flavus* using disc diffusion assay. It was found that antifungal activity was highest, with EO of *C. reticulata* where inhibition zone (IZ) was 26.1 ± 1.20 mm and MIC of 1 mg/mL for *C. albicans,* while IZ of 43.1 ± 1.67 mm and MIC of 0.25 mg/mL for *A. flavus* [131].

### 4.2. Applications of CEO for Food Packaging and Preservation

Food packaging is designed to serve the purpose of protecting the food from environmental conditions of humidity, light, and temperature, as well as others factors like dust, microorganisms, shocks, and vibrations [137,138], thereby enhancing the quality and shelf-life of foods [139]. The critical aspects of food packaging lie with the preservation of the original organoleptic properties of foods. In this regard, active packaging has emerged to extend the shelf-life of foods. Active packaging can contain intended components to be released into the foods to enhance the organoleptic properties and shelf-life, as well as to ensure food safety [140,141,142,143].

#### 4.2.1. CEO-Based Edible Films and Coatings

Recent trends in packaging show the use of biodegradable materials to reduce the load on the environment, as they are ecofriendly and nontoxic with desirable physicochemical properties over their synthetic counterparts [144]. Proteins, lipids, and polysaccharides are usually utilized to produce bio-based packaging materials for food applications [145]. Among these, proteins are extensively used because of their abundance and better film-forming property. The protein-based films exhibit excellent barrier properties for gases (e.g., O_2_ and CO_2_), as well as for volatile compounds [146]. Edible films and coatings are forms of active packaging for food preservation and shelf-life extension. These are made up of polysaccharides, proteins, and lipids that act as barriers to moisture, carbon dioxide, oxygen, and vapor [147]. Further, the edible films and coatings with antioxidant and antimicrobial properties can prevent food spoilage by microorganisms [67,69,148,149,150]. As CEOs are generally recognized as safe (GRAS) by the U.S. Food and Drug Administration (USFDA) [151], they can be used with edible films and coatings to their limits that they provide maximum protective effects without much impact on the sensory and organoleptic properties of the food [152].

Therefore, CEOs possess immense potential for their applications in food safety, packaging, and preservation. The addition of CEOs into gelatin films provided antimicrobial activity along with enhanced physicochemical properties. The incorporation of EO with chitosan, i.e., chitosan-Eos, further enhanced the properties of chitosan. The CS-EO composite edible coatings were able to extend the shelf-life of sweet pepper [153] and table grapes [154]. Further, modified chitosan coatings containing limonene (and/or EO) showed effective activity in cold-stored strawberries [155]. In addition to this, chitosan–lemon EO composite coatings exhibited antimicrobial activity and enhanced the postharvest quality of cold-stored strawberries [156]. Alparslan et al. [157] studied the effect of edible gelatin-coating enriched with orange leaf EO on the shelf-life of cold-stored pink shrimp. The addition of 2% orange leaf EO to the gelatin solution was found to extend the shelf-life of shrimps by 10 days more as compared to the noncoated control samples. Later, Alparslan and Baygar [158] studied the effect of chitosan films containing orange peel EO on the shelf-life of pink shrimp. Chitosan with 2% orange peel EO inhibited the lipid oxidation and microbial growth, thereby extending the shelf-life of shrimps by nearly eight days compared to the noncoated shrimps. Further, Randazzo et al. [159] evaluated the antimicrobial activity of eight EOs extracted from the fruit peel of the lemon, orange, and mandarin against 76 strains of *Listeria monocytogenes*. The antibacterial effect of the EOs showed the highest inhibition when incorporated into chitosan- or methylcellulose-based biodegradable films. Therefore, chitosan films with lemon EOs can be used to control *L. monocytogenes* in refrigerated conditions. Similarly, the effects of lemon, orange, and grapefruit Eos added to sodium alginate edible coating was studied on the shelf-life of fresh-cut Jintao kiwifruits by Chiabrando and Giacalone [160]. The coating with these EOs significantly inhibited the growth of yeast and mold. On the other hand, the raspberries coated with alginate and lemon EO (0.2%) or orange EO (0.1%) inhibited the growth of bacteria, yeast, and mold and showed improved post-harvest quality [161]. Also, the starch films containing orange EO were found to be effective against *L. monocytogenes* and *S. aureus* [162]. Furthermore, the effects of the nanoclay film (prepared using a solution of sodium alginate and solid sodium gelatin) with 0.4% and 0.6% of citrus essential oil were effective in controlling *Listeria monocytogenes*, *Vibrio parahaemolyticus*, *Streptococcus iniae,* and *Salmonella typhi* in fishery products [163].

#### 4.2.2. CEO-Based Microencapsulation

Microencapsulation is a technique of protecting natural ingredients, polyphenols, volatile compounds, EOs, enzymes, and even bacteria against nutritional losses and from environmental factors by encapsulating using an appropriate coating material [164]. The active component inside the microcapsule is variably known as the core, encapsulant, internal phase, payload phase, or fill, whereas the coating wall is referred as the carrier material, wall material, encapsulating agent, membrane shell, external phase, or matrix [165]. The microcapsules’ size range between 3–800 mm in diameter, and 10%–90% of their core material should remain within the capsule for a specific period before its release in foods or the target site in a controlled manner [166]. Microencapsulation can be performed by coacervation or by a drying process. The coacervation can be either in the aqueous phase (i.e., encapsulation of water-insoluble/hydrophobic core materials) or in the organic phase (i.e., encapsulation of water-soluble compounds). On the other hand, the drying process can be achieved either by fluidized-bed coating, spray-drying, spray-bed-drying, or freeze-drying (lyophilization) [166,167].

The encapsulation of EOs as core material ensures their stability by protecting direct reaction with food matrices. Therefore, the selection of appropriate coating materials, as well as the encapsulation technique, is crucial to maximizing the incorporation and retention of the functional components within the encapsulation matrix. For example, maltodextrin is a common and low-cost polysaccharide with neutral taste and aroma used as an efficient coating material for rosemary EO [168]. Further, the encapsulated phenolic compounds present in CEOs can protect foods like meat, fish, and processed products from microbial spoilage, as well as from the lipid oxidative degradation. Raksa et al. [169] prepared the kaffir lime peel EO microcapsules using a complex coacervation method with gelatin and gum arabic as wall materials and studied their antibacterial activity. The microcapsules containing the EO showed antibacterial activity against *Staphylococcus aureus*. Further, de Araújo et al. [170] performed microencapsulation of sweet orange EO (SOEO) using maltodextrin and gelatin as wall materials in different ratios. The SOEO microcapsules showed antibacterial and antioxidant properties, thereby suggesting microencapsulated CEOs to be of great importance for the food industries. The mechanism of antimicrobial action of CEOs occurs by the controlled release of the lipolytic components in CEOs and disruption of the cell membrane structure of the microbes. In addition to this, CEOs also add taste and flavor to foods [166].

#### 4.2.3. CEO-Based Nanoemulsion

The size of CEO-based nanoemulsion droplets ranges between 20–200 nm. Nanoemulsion droplets have a larger surface area and have more effective antimicrobial actions compared to microemulsion due to the larger number of droplets. However, the type and composition of feed material have to be optimized in order to obtain kinetically stable nanoemulsions [171]. Recently, hydrodynamic cavitation has emerged as a low-cost, effective, efficient, and scalable method and technology for extracting and nanoemulsifying CEOs, which also takes advantage of the copresence of pectin in citrus peels [40]. CEOs are cost-effective, environmentally friendly, and relatively nontoxic materials of interest with huge potential bioactive compounds that can be utilized through nanoemulsions for their applications in food and beverage industries. For example, the citrus (orange, grapefruit, mandarin, and lemon) essential oil-based nanoemulsions were investigated for their antioxidant and antimicrobial effects on rainbow trout fillets stored at refrigerated condition [172]. The nanoemulsions were able to restrict the growth of bacteria compared to the control group and could therefore be utilized for preserving other foods as well.

The mode of action of EOs against microbes depends on the biochemical profile and their ratio in crude EOs. Generally, the EOs disrupt the cellular structure of microbes by biochemical interactions with the cell membrane and cytoplasmic content leading to cell death [22,173,174]. Besides this, nanocarrier materials, such as alginate, cellulose, chitosan, cyclodextrin, dextran, and starch, can also alter membrane potential and the metabolic process, along with the generation of reactive oxygen species (ROS) similar to EOs for antimicrobial activity [175]. The greater surface area of nanoencapsulated EOs due to their small size provides an advantage for efficient interaction with microbial cell membranes at optimal doses [176]. Further, the delivery and release of the EOs at the targeted sites make them more effective against any microorganism [173]. Also, the EOs may act synergistically with carrier agents possessing antimicrobial properties, hence leading to the enhanced the antimicrobial activity of the nanoencapsulated EOs [177]. The applications of CEOs for food packaging and preservation are summarized in Table 5.

## 5. Future Concerns and Perspectives

The application of CEOs for food packaging and preservation eliminates the need for synthetic preservatives. However, there are certain concerns regarding the use of CEOs. For example, CEOs can be used in meat and fish for preservation, but the active volatile compounds present in CEOs may interact with the proteins and produce undesirable compounds [166]. Further, the knowledge on how EOs interact with other components within food matrices and with other antimicrobial compounds is vital for food safety [189]. If a large amount of CEO is used to ensure the preservation, then they might alter the taste and aroma of food and ultimately the quality and consumer acceptance, since CEO provides a better taste and aroma at a low concentration. Furthermore, CEOs are nontoxic, hypoallergenic, and safe for consumption, but have caused skin irritation and allergies in some cases. However, this issue could be addressed by the microencapsulation of CEOs with suitable biodegradable coating materials that would provide an efficient controlled release of the biologically active compounds to the target site. This will also address the concerns associated with CEOs’ instability and their interactions with food matrices (e.g., proteins), and further ensures that the biological activities of CEOs are unaltered through controlled release. In addition to this, the amount and composition of CEOs for their use in different food matrices are very crucial for food packaging and preservation aspects. Therefore, future research should focus on the efficient, affordable, and “green” extraction methods of CEOs, along with the optimum dose and safe limits, their interactions with various food matrices and packaging materials, possible allergic reactions, and their impacts on food quality and safety. Besides this, the use of biotechnology approaches involving genetic engineering should be utilized to develop disease-resistant *Citrus* plants in the first place [190].

## 6. Conclusions

CEOs are economic, ecofriendly, and natural alternatives to synthetic preservatives for food safety, packaging, and preservation. CEO-based edible films and coatings, microencapsulated biodegradable polymers, and nanoemulsion coatings can be applied for food packaging and preservation with enhanced antimicrobial properties. CEOs have the potential to reduce environmental pollution, substitute synthetic antimicrobials, and utilize byproducts of *Citrus* species in food processing industries. In addition, the antifungal properties of CEOs can be useful for the post-harvest disease control of fruits and vegetables in the agricultural industry. However, there are certain concerns regarding the optimum dose and safe limits, interactions with various food matrices and packaging materials, possible allergic reactions, and various advanced methods of encapsulating CEOs for efficient controlled release. These aspects require extensive and in-depth future research to ensure food safety and security.

## Figures and Tables

**Table 1:** Extraction and detection methods of essential oils (EOs) from various *Citrus* species.

*Citrus* Species	Parts Used for Extraction/Detection	Extraction Method	Purification/Detection Method	References
*Citrus limon*, *C. aurantifolia*, *C. sinensis*, *C. paradisi*	Peels	Microwave hydrodiffusion and gravity	GC-MS	[36]
*Citrus aurantium (Aurantii fructus immaturus*, *Aurantii fructus)*	Dried immature fruits, dried ripe fruits	Steam distillation (SD)	GC-FID, GC-MS	[44]
*Citrus reticulata*	Peel, leaf	Hydrodistillation (HD)	GC-MS, NMR	[45]
*Citrus aurantium*	Fruit cortex	Cold pressing	GC-MS	[46]
*Citrus aurantium*	Bud	Reflux extraction (RE), steam distillation extraction (SDE), and ultrasound-assisted extraction (UAE)	GC-MS	[47]
*Citrus limon*	Commercial oil	Brown oil extraction	GC-FID/MS, FT-MIR, H-NMR, UHPLC-TOF-MS	[48]
*Citrus limon*	Essential oil	Brown oil extraction	GC-FID/MS, FT-MIR, H-NMR, UHPLC-TOF-MS	[49]
*Citrus aurantium*	Flower	HD and ultrasonic-assisted headspace solid phase micro-extraction (UA-HS-SPME)	GC-MS	[50]
*Citruslimon*, *C. Sinensis*, *C. reticulata*, *C. paradisi*	Air dried peel	HD	GC-MS	[51]
*Citrus sinensis*, *Citrus x paradisi*, *Citrus deliciosa*, *Citrus limon*, *Citrus aurantifolia*	Essential oil	HD	GC-FID, ATR-FTIR, NIR-FT Raman	[52]
*Citrus aurantium*	Peels	SD	HS-GC-MS	[53]
*Citrus sinensis*, *Citrus limonum*, *Citrus aurantium*	Peels	HD	GC-MS	[54]
*Citrus aurantium*	Peel, flowers, and leaves	HD	GC-FID, GC-MS	[55,56,57,58]
*Citrus aurantium*	Flower	SD, Liquid–liquid extractions with n-hexane	GC/FID, GC-MS, GC/MS-LRI	[59,60]
*Citrus sinensis*	Peels	HD, Instant Controlled Pressure Drop Technique (DIC), UAE, Solvent Extraction (SE)	GC-MS	[61]
*Citrus limon*	Fruits, peels	Cold pressing, HD	GC-MS	[62]
*Citrus sinensis*, *Citrus limon*, *Citrus reticulata*, *Citrus aurentium*	Peels	SD and microwave assisted steam distillation (MASD)	GC-MS	[63,64]

**Table 2:** Major compounds of different CEOs and functions.

Compounds	Class	Plant Parts Analyzed	*Citrus* Species	Functions	References
Limonene	Monoterpene	Flower, peel, leaf	Mandarin, orange, pummelo, lemon	Anti-inflammatory, antidiabetic, anticancer, antioxidant, lipid lowering	[75,76,77]
*α*-pinene	Monoterpene	Flower, peel, leaf	Mandarin, orange, pummelo, lemon	Antimicrobial	[76,78,79]
Camphene	Monoterpene	Peel, leaf	Lemon, lime, pummelo, mandarin, sour orange	Lipid lowering	[80,81]
*β*-pinene	Monoterpene	Flower, peel, leaf	Pummelo, mandarin, orange, lemon	Antifungal	[78,82]
Sabinene	Monoterpene	Flower, peel, leaf	Pummelo, mandarin, orange, lemon	Antifungal	[75,76,78]
*α*-phellandrene	Monoterpene	Peel, leaf	Mandarin, orange	Insecticidal activity	[75,83]
*β*-myrcene	Monoterpene	Flower, leaf	Pummelo, mandarin, orange, lemon	Antifungal, embryofoetotoxicity	[76,78,84]
(E)/(Z)-ocimene	Monoterpene	Flower, peel, leaf	Pummelo, mandarin, orange, lemon	Antiviral, antifungal, anti-inflammatory, antibacterial, anti-oxidative, antiseptic effects	[75,76,78]
Valencene	Sesquiterpenes	Fruit, peel	Blood orange, *Citrus limon* L	Anti-inflammatory, anti-allergic effects	[71]
*β*-/*δ*-elemene	Sesquiterpenes	Flower, peel, leaf	Pummelo, orange, lemon, grapefruit	Anti-glioblastoma, anticancer	[76,78,85]
*α*-/*β*-copaene	Sesquiterpenes	Flower, peel	Pummelo, mandarin, orange, lemon	Attractant for male fruit flies	[76,78,86]
(E)-/*β*-caryophyllene	Sesquiterpenes	Flower, peel, leaf	Pummelo, mandarin, orange, lemon	Antimicrobial, anti-inflammatory, antibiotic, anticancer, antioxidant	[76,78,87]
*α*-humulene	Sesquiterpenes	Peel	Pummelo, mandarin, orange, lemon	Anticancer	[75,76,87]
Geranial	Sesquiterpene aldehyde	Flower, leaf	Mandarin, orange, lemon	Antifungal	[76,78,88]
Geraniol	Terpene alcohol	Flower, leaf	Pummelo, mandarin, orange, lemon	Antimicrobial, anti-inflammatory, anticancer, antioxidant	[76,78,89]
*α*-/*β*-citronellol	Monoterpene alcohol	Flower, leaf	Orange, lemon, mandarin	Anti-inflammatory, increase perspiration	[76,78]
Neral	Monoterpene aldehyde	Leaf, peel	Lemon	Antifungal	[76,88]
Nerol	Monoterpene alcohol	Flower, leaf	Orange, lemon, mandarin, pummelo	Antimicrobial	[76,78,90]
*α*-terpineol	Monoterpene alcohol	Leaf, peel	Lemon	Antifungal	[76,82]
Linalool	Monoterpene alcohol	Flower, peel, leaf	Orange, lemon, mandarin, pummelo	Antidiabetic	[75,76,78,91]
Thymol	Monoterpene phenol	Flower	Orange, mandarin,	Antimicrobial	[78,92]

**Table 3:** Antibacterial properties of CEOs and their components.

CEOs/Components	Microorganisms	Type of Microorganism	Effects	References
Orange, bergamot, lemon EO (Limonene)	*Arcobacter butzleri*	Gram −ve	MIC > 4% (*v/v)*	[117]
	*Escherichia coli (E. coli)*, *Klebsiella pneumoniae*	Gram −ve	MIC 0.6–5 mg/mL	[118]
	*Mycoplasma pneumoniae*, *Mycoplasma fermentans*	Gram −ve	MIC 0.03–1% (*v/v*)	[119]
	*Brochotrix thermospacta* NCTC 10822	Gram +ve	MSC 1.68 mg/L. 1 M solution in methanol increases UFAs	[120]
Orange, bergamot, lemon EO (Linalool)	*Arcobacter butzleri*	Gram −ve	MIC 0.06, 0.125, 0.25% (*v/v*)	[117]
	*Staphylococcus epidermidis*	Gram +ve	MIC 0.6–5 mg/mL	[118]
Orange, bergamot, lemon EO (Citral)	*Campylobacter jejuni*	Gram −ve	Antimicrobial effects 0.03–0.06% (*v/v*)	[121]
	*Staphylococcus aureus*	Gram +ve	Antimicrobial effects 0.03–0.06% (*v/v*)	[121]
*Citrus aurantium* EO	*E. coli*	Gram −ve	20.1 ± 0.56 mm (zone of inhibition with 160 µg/disc)	[122]
	*S. Typhimurium*	Gram −ve	18.2 ± 0.79 mm	
	*S. aureus*	Gram +ve	21.53 ± 0.45 mm	
	*B. cereus*	Gram +ve	20.4 ± 0.78	
	*L. monocytogenes*	Gram +ve	20.8 ± 0.53 mm	
*Citrus limon* EO	*E. coli*	Gram −ve	MIC > 6.4 mg/mL	[123]
	*Bacillus subtilis*	Gram +ve	MIC > 12.8 mg/mL	[123]
*C. reticulata* var. Blanco EO	*E. coli*	Gram −ve	16 mm inhibition zone at 9 µL/mL	[124]
Orange, bergamot, lemon EO	*Arcobacter butzleri*	Gram −ve	MIC > 4% (*v/v*)	[117]
Orange, bergamot, lemon EO	*Klebsiella pneumoniae*	Gram −ve	MIC 0.00125–0.050 mL/mL	[125]
	*Staphylococcus aureus*	Gram +ve		

**Table 4:** Antifungal properties of CEOs and their components.

Fungi	CEOs	Components	Tests	Effects	References
*Aspergillus niger (A. niger)*	Lemon (*C. limon* Burm.f.)	Citral, eugenol	Agar diffusion method (ADM)	inhibition halo = 10 mm	[18]
*A. flavus*	Orange (*C. sinensis* Osb.)	Limonene, citral	Poisoned food assay (PFA)	100% inhibition at 750 ppm	[132]
*A. fumigatu*	Orange (*C. sinensis* Osb.)	Limonene, citral	PFA	100% inhibition at 750 ppm	[132]
*A. terreus*	pummelo (*C. maxima* Burm.)	Limonene, citral	PFA	100% inhibition at 750 ppm	[132]
*A. parasiticus*	Lime (*C. hystrix* DC)	limonene, citronellol, linalool	ADM; broth microdilution	MIC = 1%, 4% (*v/v*)	[20]
*Penicillium chrysogenum (P. chrysogenum)*	Orange (*C. sinensis* Osb.)	Limonene, myrcene	ADM; broth microdilution	Inhibition zone = 18.99 mm; MIC = 9.33 μL/mL	[133]
*P. digitatum*	Orange (*C. sinensis* Osb.)	Limonene, myrcene	Dry weight determination	ED_50_ = 2180.2 ppm (A1)	[134]
*P. italicum*	Mandarin (*C. reticulata* Blanco)	Limonene, *γ*-terpinene	Poisoned food technique	100% inhibition at 2.5 μL/mL	[84]
*P. expansum*	Orange (*C. sinensis* Osb.)	Limonene, myrcene	ADM	34.9% inhibition at 2000 ppm	[135]
*Fusarium oxysporum (F. oxysporum)*	Mandarin (*C. reticulata* Blanco)	Limonene, geranial	PFA; volatile activity assay (VAA)	MIC > 0.2% (*v/v*)	[130]
*F. proliferatum*	Lime (*C. autantifolia*)	Limonene, *β*-pinene	ADM	91.5% inhibition at 2000 ppm	[135]
*Alternaria alternata*	Mandarin (*C. reticulata* Blanco)	Limonene, geranial	PFA; VAA	MIC = 0.2% (*v/v*)	[130]
*Rhizoctonia solani*, *Curvularia lunata*	Mandarin (*C. reticulata* Blanco)	Limonene, geranial	PFA; VAA	MIC = 0.2% (*v/v*)	[130]
*Rhizopus* spp.	Lemon (*C. limon* Burm.f.)	Citral, eugenol	ADM	Inhibition halo = 12 mm	[18]
*Helminthosporium oryzae*	Mandarin (*C. reticulata* Blanco)	Limonene, geranial	PFA; VAA	MIC > 0.2% (*v/v*)	[130]
*Botryodiplodia theobromae*, *Myrothecium roridum*	Orange (*C. sinensis* Osb.)	Limonene, linalool	PFA; VAA	MIC = 600 ppm/ 700 ppm (VA)	[136]
Mucor hiemalis	Orange (*C. sinensis* Osb.)	Limonene, myrcene	ADM	36.5% inhibition at 2000 ppm	[135]
*Helminthosporium oryzae*, *Trichoderma viride*	Orange (*C. sinensis* Osb.), pummelo (*C. maxima* Burm.)	Limonene, citral	PFA	100% inhibition at 750 ppm	[132]

**Table 5:** Application of CEOs for food packaging and preservation.

CEO/Formulation	Packaging	Preservation	References
Bergamot EO	Chitosan and hydroxypropylmethyl cellulose (HPMC) edible films	Antibacterial activity against *Escherichia coli*, *Listeria monocytogenes* and *Staphylococcus aureus*	[178]
Bergamot EO	Gelatin edible films	Antibacterial activity against *Staphylococcus aureus* and *Listeria monocytogenes*	[179]
Bergamot and bitter orange EOs	Chitosan and locust bean gum edible films	Effective against *Aspergillus flavus* in dates	[180]
Bergamot, lemon and mandarin EOs	Modified chitosan nanoemulsion coating	Antimicrobial activity against *Escherichia coli* O157:H7 and *Samonella typhimurium*	[181]
Bergamot EO	Chitosan coatings	Protect oranges from *Penicillium italicum*	[182]
Lemon EO	Novel edible coating with modified chitosan and nanoemulsified lemon EO	Increases antimicrobial activity and prolongs the shelf life of vegetable products	[183]
Lemon EO	Incorporated on to the modified chitosan edible coating	Protects the storage-keeping quality strawberries	[156]
Lime EO	Chitosan-based edible coating	Inhibition of *Rhizopus stolonifera* in fresh tomato	[184]
Lime EO	Gum-based edible coating	Reduction of *Colletotrichum gloeosporioides* and *Rhizopus stolonifer* in fresh papaya	[127]
Lemon EO	Low density polyethylene (LDPE) films	Acts as flavoring films for packaging biscuit, prevents changes in water-vapor permeability and mechanical properties	[185]
Lemon EO	Chitosan	Delayed ripening with a lower respiration rate was observed in strawberries coated with lemon EO-based chitosan coatings	[186]
Lemon EO	Chitosan	Chitosan film combined with lemon, thyme and cinnamon essential oils provide a new formulation for antimicrobial films	[187]
*Citrus reticulata* var. tangerine EO	Nanoemulsions based on chitosan nanoparticles	Effective in preventing microbial growth and lipid oxidation in silvery pomfret and shows the potential to preserve seafoods	[188]
